# Partial replacement of soybean meal with *Musca domestica* larvae meal in broiler diets: implications for growth performance, nutrient utilization, hemato-biochemical profile and organoleptic characteristics

**DOI:** 10.5194/aab-67-247-2024

**Published:** 2024-06-14

**Authors:** Momin Khan, Naila Chand, Sarzamin Khan, Shabana Naz, Abdulwahed Fahad Alrefaei, Ananthanarayanan Chandrasekaran, Rifat Ullah Khan

**Affiliations:** 1 Department of Poultry Science, Faculty of Animal Husbandry and Veterinary Sciences, The University of Agriculture, Peshawar, Pakistan; 2 Department of Zoology, Government College University, Faisalabad, Pakistan; 3 Department of Zoology, College of Science, King Saud University, P.O. Box 2455, Riyadh 11451, Saudi Arabia; 4 Department of Physics, Sri Sivasubramaniya Nadar College of Engineering, Kalavakkam-603110, Tamil Nadu, India; 5 College of Veterinary Sciences, Faculty of Animal Husbandry and Veterinary Sciences, The University of Agriculture, Peshawar 25000, Pakistan

## Abstract

The present investigation aimed to assess the impact of substituting soybean meal (SBM) with *Musca domestica* larvae meal (HMM) in the diet of broilers (Ross 308, n=1000 ) during the initial 1–28 d of their growth period. Four isocaloric and isonitrogenous broiler rations were formulated, including a control group (Mag-0) with 100 % SBM and 0 % HMM, diet 2 (Mag-10) with 90 % SBM and 10 % HMM, diet 3 (Mag-20) with 80 % SBM and 20 % HMM, and diet 4 (Mag-30) with 70 % SBM and 30 % HMM. The analysis of amino acid concentrations in diets revealed slight increases in most essential and nonessential amino acids, except for phenylalanine, arginine, glutamic acid, aspartic acid and serine, with increasing levels of SBM substitution with HMM. Digestibility of nutrients, including dry matter, nitrogen-free extract, crude protein ether extract, ash and crude fiber, was not significantly affected across different substituted diets. Similarly, amino acid digestibility did not differ among various diets of SBM substituted with house fly larvae meal. Weekly and overall body weight gain, feed intake and feed conversion ratios were similar across all birds fed different replacement diets. Apparent metabolizable energy, protein efficiency ratio, dressing percentage and antibody titre also showed no significant differences among the substituted diets and the control group. Similarly, hematological and organoleptic studies exhibited statistically similar effects. Overall, the study concludes that substituting up to 30 % of SBM with HMM in broiler rations does not adversely affect health or performance in broiler chickens.

## 1 Introduction

In poultry nutrition, recent advances have focused on exploring and utilizing more affordable alternative protein sources to enhance poultry health and production (Laudadio et al., 2012; Saad et al., 2023). Feed represents a significant cost in broiler production, especially considering the fact that similar feed ingredients are used for both human and avian consumption (Dhama et al., 2015). Feed constitutes over 60 % of the total expenditure in the poultry industry; thus, prioritizing the development of economically viable and premium diets is crucial (Khan et al., 2010). This approach underscores the necessity of substituting locally available feed ingredients, encompassing both traditional and unconventional options (Orinetha et al. 2022). With feed resources becoming increasingly scarce and with prices rising, the poultry feed industry is under severe demand to improve feed efficiency. Traditionally, soybean meal (SBM) has played a vital role as a protein source in poultry nutrition. Nonetheless, diverse challenges such as trade disputes, limited availability, intermittent supply, political interference and high costs hinder its consistent use (Yue et al., 2023). In order to produce cost-effective poultry feed, it is essential to identify efficient animal-origin high-quality protein sources (Elahi et al., 2019).

**Table 1:** Ingredient and chemical composition (%) of experimental rations for broiler birds for 1–14 d.

Ingredients	Diets (soybean meal : maggot meal)			
	Mag-0	Mag-10	Mag-20	Mag-30
Corn	54	54	54	54
Sunflower meal	10	10	10	10
Rice polish	6	6	6	6
Cotton seed meal	2.3	2.3	2.3	2.3
Molasses	1	1	1	1
Soybean meal	10	9	8	7
Maggot meal	0	0.82	1.64	2.46
Maize gluten meal	3.5	3.5	3.5	3.5
Fish meal	5	5	5	5
Brocken rice	2.58	2.58	2.76	2.94
Guar meal	4	4	4	4
Limestone	0.4	0.4	0.4	0.4
Lysine	0.1	0.1	0.1	0.1
Rock phosphate	1	1	1	1
Methionine	0.1	0.1	0.1	0.1
Mineral vitamin premix	0.1	0.1	0.1	0.1
Salt	0.1	0.1	0.1	0.1
Calculated chemical composition				
Dry matter (%)	89.3	89.5	89.8	90.1
Metabolizable energy (kcal kg ${}^{-1}$ )	2987	2994	2998	3010
Crude protein	21.3	21.4	21.1	21.2
Ash	5.68	5.74	5.79	5.87
Ether extract	4.18	4.34	4.41	4.49
Fiber	4.34	4.31	4.32	4.31
Calcium	0.74	0.75	0.78	0.79
Phosphorous	0..34	0..35	0.36	0.38
Lysine	1.32	1.34	1.35	1.36
Cysteine	0.36	0.37	0.39	0.4
Methionine	0.49	0.56	0.59	0.63

**Table 2:** Ingredient and chemical composition (%) of experimental rations for broiler birds from 15 to 28 d.

Ingredients	Diets (soybean meal : maggot meal)			
	Mag-0	Mag-10	Mag20	Mag30
Corn	58.7	58.4	58.4	58.5
Brocken rice	2.1	2.7	2.5	2.8
Cotton meal	4	4	4	4
Guar meal	3.9	3.8	3.9	3.8
Sunflower meal	2.2	2.3	2.3	2.3
Soybean meal	10	9	8	7
Maggot meal	0	0.82	1.64	2.46
Maize gluten meal (30 %)	7.5	7.4	7.4	7.5
Fish meal 50 %	3.9	3.9	3.9	3.8
Rice polish	5	5	5.3	5.2
Molasses	1	1	1	1
Lime stone	0.4	0.4	0.4	0.4
Rock phosphate	1	1	1	1
Lysine	0.1	0.1	0.1	0.1
Methionine	0.1	0.1	0.1	0.1
Mineral vitamin premix	0.1	0.1	0.1	0.1
Metabolizable energy (kcal kg ${}^{-1}$ )	3021	3025	3028	3029
Calculated chemical composition				
Crude protein	20.1	20.3	20.5	20.6
Ether extract	4.1	4.21	4.24	4.28
Fiber	3.93	3.94	3.92	3.94
Ash	5.44	5.45	5.44	5.47
Calcium	0.78	0.76	0.77	0.77
Phosphorous	0.3	0.31	0.3	0.32
Lysine (% of total amino acids)	1.36	1.37	1.38	1.39
Methionine (% of total amino acids)	0.46	0.58	0.64	0.7
Cysteine (% of total amino acids)	0.34	0.33	0.33	0.33

Common housefly larvae (*Musca domestica*) have become recognized as a valuable protein source for broilers, offering high-quality nutrition. Their nutritional content and amino acid composition exceed those of commonly used feed ingredients like soybean meal and fish meal. Therefore, *Musca domestica* larvae meal (HMM) presents the opportunity to replace expensive protein sources in poultry feed formulations, both from an environmental and economic perspective (Khan et al., 2016; M. Khan et al., 2018). Earlier investigations have explored the feasibility of incorporating HMM into broiler diets, resulting in diverse findings. Khan et al. (2016) reported improved growth performance and enhanced organoleptic properties of meat when 30 % of SBM was replaced with HMM. Similarly, S. Khan et al. (2018) incorporated HMM by up to 60 %, yielding positive results in terms of growth performance. On the contrary, Elahi et al. (2019) substituted HMM for SBM at an 8 % rate, observing no significant effects on growth performance or immune response in broilers. However, a study by Murawska et al. (2021) investigated the replacement of black soldier fly meal with soybean meal at relatively higher inclusion rates (50 %, 75 % and 100 %), revealing diminished growth performance and significantly reduced taste and juiciness in broiler meat at higher inclusion levels. To date, no study has reported the complete replacement of SBM with HMM in broiler rations. Therefore, this study aims to replace SBM with HMM at rates of 10 %, 20 % and 30 % and to assess the impact of such on growth performance, immune response, and hematology and serum analytes in broilers.

## 2 Materials and methods

### 2.1 Processing of HMM

The HMM was prepared according to the method described by Reda et al. (2023). Initially, intact poultry guts were obtained from animals slaughtered at a local abattoir. These gut samples were exposed to houseflies for at least 3 h and were then submerged in water for about 1 week to allow the larvae to fully develop. Once matured, the maggots were harvested, separated from the gastrointestinal tract contents, euthanized with hot water (70 °C for 60 min), sun-dried (35 ± 2.4 °C for 3 h daily for a total period of 7 d) and ground into a fine powder using a micro-mixer. To prevent microbial contamination, the dried maggots underwent a brief heating process in a hot oven before being included in the feed formulation.

### 2.2 Fatty acid composition of maggots

Briefly, a 1 g sample underwent extraction using a chloroform–methanol mixture ( 2:1 ) following the protocol outlined by Folch et al. (1957). Subsequently, the extracted fat was transformed into fatty acid methyl esters using boron trifluoride in methanol (Wang et al., 2013). Gas chromatography (HP7890 series gas chromatograph, Agilent Technologies, Santa Clara, CA), fitted with a flame ionization detector, was then employed to separate and analyze the obtained fatty acid methyl esters. The column used was 60 m × 0.25 mm internal diameter × 0.25 µ m film thickness.

### 2.3 Amino acid analysis of maggots 

At the PCSIR lab in Peshawar, Pakistan, the amino acid content of maggot samples was analyzed using high-performance liquid chromatography (HPLC) using the Applied Biosystems 3200 Q TRAP LC/MS/MS system, equipped with an RP-C18 column (100 mm × 2.1 mm, 1.7 µ m), with techniques by Ahmad et al. (2022). The process involved hydrolyzing finely crushed materials with barium hydroxide and boiling water, adjusting the pH with HCl, and diluting the solution. Acid hydrolysis with HCl at high temperatures was conducted, followed by vacuum drying and dissolution in citrate buffer. The derivative was injected into the HPLC, where a Shimadzu (Shimadzu Oceania, Pickering Laboratories, Mountain View, CA, USA) detector was used with a binary gradient technique for amino acid resolution. Two solvents were pumped into the column at a specific rate for a set duration.

### 2.4 Birds and husbandry

During the 1–28 d period, male broiler chickens ( n=1000 , Ross 308) were raised and divided into four dietary groups. Each group comprised 10 pens, with 25 birds housed in each pen. The pens were sized at 1.25 × 1.35 m, equipped with manual feeders, drinkers and sawdust bedding material. Heat lamps were utilized during the initial 3 weeks to maintain the required temperature for the birds. The lighting schedule remained consistent, with 20 h d ${}^{-1}$ throughout the experiment. The birds had access to feed and water ad libitum throughout the entire trial period.

### 2.5 Preparation of experimental diets

The diets were prepared with graded levels of HMM (10 %, 20 % and 30 %) on an as-fed basis. The experiment was formulated to meet the need of the starter (1–14 d) and finisher (15–28 d) phases specified in Tables 1 and 2. The control diet (Mag-0) contained SBM (48 %) as the main protein source. In the experimental diets, the protein from SBM was gradually substituted with different levels (10 %, 20 % and 30 %) of protein from HMM (referred to as Mag-10, Mag-20 and Mag-30, respectively). The amino acid and fatty acid composition of HMM are presented in Table 3.

**Table 3:** Fatty acid and amino acid composition of maggot meal.

Fatty acid	g per 100 g	Amino acids	g per 100 g
Myristic acid (C14:0)	15.23	Arginine	3.2
Palmitic acid (C16:0)	11.23	Histidine	2.6
Palmitoleic acid (C16:1n7)	2.8	Isoleucine	2.32
Stearic acid (C18:0)	12.32	Leucine	6.98
Oleic acid (C18:1N9)	5.4	Lysine	4.2
Linoleic acid (c18:2n6)	0.6	Methionine	4.1
Docosahexaenoic acid (c22:6)	1.2	Phenylalanine	1.6
Eicosapentaenoic acids (c20:5 omega 3)	0.9	Threonine	2.4
Linolenic acids (c18:3omega 6)	1.2	Valine	2.89
Heptadecanoic acids (c17::0)	24	Alanine	3.25
Lauric acids (c12:0)	3.4	Aspartic acid	4.47
Capric acids (10:0)	0.05	Cysteine	0.8
Tridecanoic acids (C13:0)	0.64	Glycine	2.45
Pentadecanoic acid (15:0)	0.5	Glutamic acid	4.45
		Proline	2.89
		Serine	2.8
		Tyrosine	2.76
		Tryptophan	0.2

### 2.6 Growth performance 

The body weight and feed intake of the broilers were randomly recorded weekly on a per-pen basis for each treatment throughout the duration of the study. Feed spoilage was mitigated through the implementation of various strategies, including proper feeder design, feed management involving feeding schedules and portion control, maintaining clean and dry feeding areas, personnel training, and regular monitoring. Feed conversion ratio (FCR) was calculated weekly and for the entire duration of the experiment to evaluate feed efficiency. A sensitive electronic scale was used to measure all measurements, including feed intake and weight gain, to ensure reliability and accuracy. The protein efficiency ratio (PER) was calculated by dividing weight gain by protein intake, following the method outlined by Kamran et al. (2008). At the conclusion of the study (28 d), three birds were randomly chosen from each pen within every feeding group and were slaughtered and eviscerated. Dressing percentage was determined for the selected carcasses, and breast and thigh weights were additionally assessed.

**Table 4:** Impact of different substitution levels of maggot meal replacement of soybean meal on the analyzed amino acid concentration (g kg ${}^{-1}$ ) in broiler chicks.

Amino acid	Mag-0	Mag-10	Mag-20	Mag-30
Isoleucine	8.34	8.39	8.85	8.97
Histidine	6.32	6.54	6.63	6.97
Methionine	4.41	4.76	5.32	5.63
Lysine	7.34	7.43	7.84	7.98
Threonine	9.52	9.87	10.2	10.4
Phenylalanine	10.2	10.1	9.87	8.79
Arginine	9.32	9.16	8,93	8.11
Leucine	19.2	19.8	20.2	20.4
Valine	9.23	9.45	9.87	10.2
Alanine	15.1	15.3	15.9	16.4
Aspartic acid	20.3	19.7	19.2	18.8
Cysteine	3.24	3.56	4.32	4.65
Glycine	9.76	10.3	11.9	12.4
Glutamic acid	34.2	32.2	31.3	30.3
Proline	17.4	17.2	19.3	16.6
Serine	15.3	14.7	14.2	13.4
Tyrosine	10.3	10.6	10.8	11.3

Mag represents maggot meal: Mag-0 is the control diet (having 100 % soybean meal and 0 % maggot meal), whereas the other diets are Mag-10 (containing 90 % soybean meal and 10 % maggot meal), Mag-20 (having 80 % soybean meal and 20 % maggot meal) and Mag-30 (having 70 % soybean meal and 30 % maggot meal).

### 2.7 Nutrient digestibility

At the conclusion of the study, the birds were euthanized humanely through cervical dislocation. These samples underwent freeze-drying to remove moisture followed by lyophilization under vacuum conditions to further dehydrate them. Subsequently, the samples were ground and prepared for analysis. The concentration of chromic oxide was assessed using a UV–Vis spectrophotometer (ISR-2600 plus), while dry matter was passed through bomb calorimetry (Chincan, model Ck-XRY-IA, Zhejiang, China) to calculate gross energy, as per the methods outlined by Nascimento Filho et al. (2021) and Kierończyk et al. (2022).

The apparent metabolizable energy (AME) values were computed utilizing a dedicated equation derived from the analysis results:

1 $$\begin{array}{rl} & \mathrm{AME}(\mathrm{kcal}\;{\mathrm{kg}}^{-1}\mathrm{of}\;\mathrm{diet})\\ & \;=\text{GE diet-}[\text{GE in digesta/excreta}\\ & \;\times \;\left(\frac{\text{marker diet}}{\text{marker excreta/digesta}}\right)], \end{array}$$

where GE refers to gross energy, and marker refers to the chromic oxide concentration.

The ground ileal samples, with a particle size of less than 0.1 mm, were utilized to determine the digestibility coefficients of nutrients. An indigestible marker, namely 0.2 % chromic oxide (Cr2O3), was employed to assess the coefficients of apparent total digestibility using the following formula:

2 $$\begin{array}{rl} & \text{Apparent digestibility }\\ & \;=100-\left[100\;\times \frac{\%\;\text{nutrient in digesta}\;\times \;\%\;\text{Cr in diet}}{\%\;\text{nutrient in diet}\;\times \;\%\;\text{Cr in digesta}}\right] \end{array}.$$

For determination of ileal amino acid digestibility, the following formula was used:

3 $$\begin{array}{rl} & \text{Apparent ileal digestibility of amino acids}\\ & \;=\;[1-\left(\frac{\text{Cr, in diet}}{\text{Cr in Digesta}}\right)\\ & \times \;\left(\frac{\text{amino acid in digesta}}{\text{amino acid in diet}}\right)\;\times \;100] \end{array}.$$

**Table 5:** Impact of different substitution levels of maggot meal replacement of soybean meal on the digestibility of nutrients in broiler chicks.

Nutrient digestibility (%)	Treatment groups				p value
	Mag-0	Mag-10	Mag-20	Mag-30	
Dry matter	80.67 ± 1.35	81.19 ± 0.83	80.83 ± 1.18	81.49 ± 1.18	0.957
Crude protein	81.39 ± 1.73	80.66 ± 0.69	80.15 ± 1.07	80.71 ± 1.05	0.906
Ether extract	72.57 ± 0.544	72.01 ± 0.78	72.27 ± 0.44	72.66 ± 0.88	0.905
Ash	43.90 ± 0.55	44.88 ± 0.82	43.82 ± 0.81	44.13 ± 0.89	0.769
Crude fiber	82.16 ± 0.93	81.63 ± 1.63	81.52 ± 0.72	80.91 ± 0.63	0.868

Mag represents maggot meal: Mag-0 is the control diet (having 100 % soybean meal and 0 % maggot meal), whereas the other diets are Mag-10 (containing 90 % soybean meal and 10 % maggot meal), Mag-20 (having 80 % soybean meal and 20 % maggot meal) and Mag-30 (having 70 % soybean meal and 30 % maggot meal).

**Table 6:** Impact of different substitution levels of maggot meal replacement of soybean meal on the total-tract amino acid digestibility coefficient in broilers.

Amino acids	Treatments				p value
	Mag-0	Mag-10	Mag-20	Mag-30	
Essential amino acids					
Isoleucine	0.85 ± 0.006	0.83 ± 0.003	0.84 ± 0.009	0.85 ± 0.006	0.517
Lysine	0.81 ± 0.006	0.82 ± 0.003	0.81 ± 0.009	0.83 ± 0.007	0.699
Arginine	0.84 ± 0.003	0.84 ± 0.006	0.85 ± 0.006	0.84 ± 0.003	0.897
Leucine	0.83 ± 0.007	0.83 ± 0.003	0.82 ± 0.007	0.83 ± 0.006	0.914
Histidine	0.78 ± 0.006	0.80 ± 0.003	0.81 ± 0.003	0.80 ± 0.003	0.413
Methionine	0.84 ± 0.003	0.84 ± 0.003	0.85 ± 0.009	0.85 ± 0.009	0.822
Phenylalanine	0.85 ± 0.003	0.85 ± 0.006	0.86 ± 0.009	0.87 ± 0.012	0.535
Threonine	0.71 ± 0.009	0.72 ± 0.013	0.74 ± 0.009	0.74 ± 0.009	0.280
Valine	0.84 ± 0.003	0.85 ± 0.012	0.87 ± 0.003	0.85 ± 0.006	0.344
Nonessential amino acids					
Alanine	0.82 ± 0.007	0.83 ± 0.006	0.84 ± 0.006	0.84 ± 0.003	0.298
Aspartic acid	0.71 ± 0.006	0.72 ± 0.003	0.74 ± 0.007	0.74 ± 0.009	0.222
Cysteine	0.83 ± 0.003	0.85 ± 0.006	0.86 ± 0.006	0.86 ± 0.012	0.343
Glycine	0.79 ± 0.007	0.79 ± 0.009	0.80 ± 0.010	0.79 ± 0.006	0.729
Glutamic acid	0.78 ± 0.009	0.79 ± 0.012	0.81 ± 0.015	0.81 ± 0.017	0.177
Proline	0.76 ± 0.003	0.79 ± 0.006	0.78 ± 0.003	0.79 ± 0.006	0.337
Serine	0.76 ± 0.000	0.77 ± 0.003	0.78 ± 0.003	0.77 ± 0.003	0.772
Tyrosine	0.86 ± 0.003	0.85 ± 0.006	0.84 ± 0.010	0.87 ± 0.013	0.605

Mag represents maggot meal: Mag-0 is the control diet (having 100 % soybean meal and 0 % maggot meal), whereas the other diets are Mag-10 (containing 90 % soybean meal and 10 % maggot meal), Mag-20 (having 80 % soybean meal and 20 % maggot meal) and Mag-30 (having 70 % soybean meal and 30 % maggot meal).

### 2.8 Serum HI antibody assay

On day 28, 1.5 mL blood samples were collected from the wing vein and centrifuged (3000 rpm for 10 min), and the serum was stored at - 20 °C. Serum dilutions were prepared in a microplate followed by the addition of phosphate-buffered saline and Newcastle disease virus (NDV) antigen. After incubation, erythrocyte suspension was added, and the endpoint was determined for geometric mean titer calculation.

### 2.9 Hematological and serum biochemical analysis 

At the trial's end, 3 mL blood samples were collected from 24 birds, 6 from each group. Samples were drawn from the wing vein using a sterile syringe. Half of each sample was treated with ethylenediaminetetraacetic acid (EDTA) and analyzed for various blood parameters using an automatic hematology analyzer (Abaxis HM5C and V2 Allied Analytic, USA).

These serum samples were later analyzed for glucose and total protein levels using commercial kits from Biocheck Inc., UK, providing insights into the birds' metabolic and nutritional statuses.

### 2.10 Organoleptic study

A sensory evaluation of broiler meat was conducted by a panel of experts, including postgraduate students and faculty members. Samples from the breast, thigh and leg portions were cooked to 82 °C and assessed individually for organoleptic parameters using a five-point hedonic scale (Khan et al., 2016).

**Table 7:** Impact of different substitution levels of maggot meal replacement of soybean meal on a weekly basis and based on the total feed intake in broilers.

Groups	Feed Intake				Total
	Week I	Week II	Week III	Week IV	
Mag-0	236 ± 3.2	427 ± 11.9	867 ± 3.0	963 ± 7.6	2493 ± 6.6
Mag-10	230 ± 3.9	441 ± 7.4	889 ± 16.3	968 ± 6.1	2527 ± 14.5
Mag-20	231 ± 2.0	415 ± 2.1	866 ± 2.4	950 ± 6.8	2462 ± 7.9
Mag-30	239 ± 4.7	424 ± 10.6	867 ± 4.7	965 ± 9.2	2495 ± 20.9
p value	0.304	0.304	0.235	0.380	0.058

Mag represents maggot meal: Mag-0 is the control diet (having 100 % soybean meal and 0 % maggot meal), whereas the other diets are Mag-10 (containing 90 % soybean meal and 10 % maggot meal), Mag-20 (having 80 % soybean meal and 20 % maggot meal) and Mag-30 (having 70 % soybean meal and 30 % maggot meal).

**Table 8:** Impact of different substitution levels of maggot meal replacement of soybean meal on a weekly basis and based on the overall total body weight gain in broilers.

Groups	Weight gain (g)				Total
	Week I	Week II	Week III	Week IV	
Mag-0	177 ± 4.1	227 ± 5.0	413 ± 4.4	429 ± 4.7	1246 ± 1.3
Mag-10	175 ± 2.4	240 ± 6.0	424 ± 4.5	429 ± 3.4	1268 ± 10.1
Mag-20	176 ± 5.9	225 ± 1.2	413 ± 7.3	426 ± 4.2	1240 ± 8.6
Mag-30	179 ± 3.1	236 ± 6.2	419 ± 5.5	436 ± 5.5	1270 ± 12.0
p value	0.877	0.178	0.476	0.502	0.099

Mag represents maggot meal: Mag-0 is the control diet (having 100 % soybean meal and 0 % maggot meal), whereas the other diets are Mag-10 (containing 90 % soybean meal and 10 % maggot meal), Mag-20 (having 80 % soybean meal and 20 % maggot meal) and Mag-30 (having 70 % soybean meal and 30 % maggot meal).

### 2.11 Statistical analysis

Data analysis was conducted using STATISTICA version 13.3 software. The Shapiro–Wilk test assessed data distribution. The pen ( n=10 ) and the individual bird ( n=20 ) served as experimental units for growth performance and carcass quality, respectively. One-way ANOVA and Tukey tests ( p≤0.05 ) identified significant differences among group means.

## 3 Results

The amino acid concentrations of various feed ingredients are outlined in Table 4. Upon reviewing the data, it is evident that all the diets employed in this experiment contain adequate levels of essential and nonessential amino acids necessary for the normal growth of broiler birds, albeit with slight variations in concentration. The levels of most studied amino acids exhibited a slight increase with the escalating inclusion of HMM as a substitute for SBM, except for arginine, phenylalanine, aspartic acid, serine and glutamic acid.

Table 5 displays the total tract nutrient digestibility of crude protein, crude fiber, dry matter, nitrogen-free extract, ash and ether extract for the various treatment groups. Upon examination of the data, it is evident that there are no significant differences ( p≥0.05 ) observed among the different treatment groups in terms of nutrient digestibility.

Table 6 illustrates the total-tract amino acid digestibility coefficients of different formulated feeds. The data indicate no significant difference ( p≥0.05 ) in the coefficients of digestibility of essential and nonessential amino acids among the various formulated diets.

The overall and weekly feed intakes of the substituted and control groups are presented in Table 7. Statistical analysis of the data reveals that different dietary groups had no significant effect on the overall and the weekly feed intake in broiler chicks.

The weekly and total body weight gains of broiler birds are depicted in Table 8. Statistical analysis of the data indicated no significant ( p>0.05 ) effect on weekly weight during the entire experimental period. Furthermore, the overall weight gain was non-significant ( p≥0.05 ) between the control and soybean-substituted groups.

Table 9 represents the weekly and total FCR of different experimental groups. It was noted that the weekly and the total FCR values were not affected ( p≥0.05 ) by replacement of SBM with HMM in the rations of broiler chicks.

Table 10 presents the PER, dressing percentage (DP), antibody titer and AME. Statistical analysis of the data indicated that the different diets had no significant effect on the protein efficiency ratio (PER). However, a linear non-significant increase in PER was recorded with increasing substitution levels of SBM with HMM. A statistically similar ( p≥0.05 ) dressing percentage was observed for all the treatment groups with various substitution levels of SBM with HMM and for the control group. Upon review of the data, no differences ( p>0.05 ) were observed for metabolizable energy among the different treatment groups, as well as in the control group. However, the observed data showed a non-significant linear increase ( p≥0.05 ) with increasing substitution levels of SBM with HMM. No significant difference ( p≥0.05 ) was observed between the control group and the groups of SBM substituted with HMM.

**Table 9:** Impact of different substitution levels of maggot meal replacement of soybean meal on FCR in broilers.

Groups	FCR				Total
	Week I	Week II	Week III	Week IV	
Mag-0	1.33 ± 0.01	1.88 ± 0.01	2.10 ± 0.02	2.24 ± 0.01	2.00 ± 0.01
Mag-10	1.32 ± 0.01	1.83 ± 0.03	2.10 ± 0.03	2.26 ± 0.02	1.99 ± 0.02
Mag-20	1.32 ± 0.03	1.85 ± 0.001	2.09 ± 0.03	2.23 ± 0.007	1.99 ± 0.02
Mag-30	1.34 ± 0.004	1.80 ± 0.02	2.07 ± 0.02	2.23 ± 0.01	1.96 ± 0.003
p value	0.825	0.064	0.825	0.378	0.353

Mag represents maggot meal: Mag-0 is the control diet (having 100 % soybean meal and 0 % maggot meal), whereas the other diets are Mag-10 (containing 90 % soybean meal and 10 % maggot meal), Mag-20 (having 80 % soybean meal and 20 % maggot meal) and Mag-30 (having 70 % soybean meal and 30 % maggot meal).

**Table 10:** Impact of different substitution levels of maggot meal replacement of soybean meal on PER, dressing percentage, apparent metabolizable energy and antibody titer in broilers.

Groups	PER	DP (%)	Breast weight ${}^{*}$	Thigh weight ${}^{*}$	AME (MJ kg ${}^{-1}$ )	Antibody titre
Mag-0	2.53 ± 0.02	64.97 ± 0.24	24.74 ± 0.21	28.65 ± 0.43	12.67 ± 0.09	5.27 ± 0.09
Mag-10	2.50 ± 0.01	65.07 ± 0.43	24.98 ± 0.91	27.99 ± 0.211	12.72 ± 0.17	5.47 ± 0.15
Mag-20	2.53 ± 0.02	64.92 ± 0.89	24.45 ± 0.88	28.34 ± 0.26	12.78 ± 0.10	5.53 ± 0.12
Mag-30	2.55 ± 0.004	64.30 ± 0.57	24.34 ± 0.32	28.55 ± 0.32	12.85 ± 0.11	5.33 ± 0.12
p value	0.450	0.780	0.550	0.98	0.738	0.431

Mag represents maggot meal: Mag-0 is the control diet (having 100 % soybean meal and 0 % maggot meal), whereas the other diets are Mag-10 (containing 90 % soybean meal and 10 % maggot meal), Mag-20 (having 80 % soybean meal and 20 % maggot meal) and Mag-30 (having 70 % soybean meal and 30 % maggot meal). ${}^{*}$ Related-part weights/body weights × 100.

The hematology and serology of different substituted and control groups are presented in Table 11. No significant differences were recorded between the different groups of SBM substituted with HMM and the control group in terms of blood glucose, total protein, red blood cells (RBCs), packed cell volume (PCV), white blood cells (WBCs), mean corpuscular hemoglobin concentration (MCHC), mean corpuscular volume (MCV) and hemoglobin (Hb). The recorded data indicate that HMM replacement of SBM in the broiler diet has no harmful effects on the health performance indicators.

The effect of the replacement of SBM with HMM on the taste quality of broiler meat is presented in Table 12. Statistical analysis of the data revealed that the addition of HMM into the diet of broilers does not affect the organoleptic parameters of the meat.

**Table 11:** Impact of different substitution levels of maggot meal replacement of soybean meal on hematological parameters in broilers.

Blood parameters	Treatment Groups				p value
	Mag-0	Mag-10	Mag-20	Mag-30	
Blood glucose (mg dl ${}^{-1}$ )	12.7 ± 0.19	12.70 ± 0.25	12.7 ± 0.30	12.8 ± 0.05	0.974
Total protein (g dl ${}^{-1}$ )	3.68 ± 0.17	3.58 ± 0.12	3.57 ± 0.11	3.55 ± 0.17	0.922
RBC (10 ${}^{6}$ mm ${}^{-3}$ )	2.76 ± 0.06	2.79 ± 0.09	2.83 ± 0.06	2.81 ± 0.06	0.896
WBC (10 ${}^{3}$ mm ${}^{-3}$ )	9.61 ± 0.13	9.69 ± 0.13	9.68 ± 0.09	9.68 ± 0.10	0.950
PCV (%)	31.6 ± 0.09	31.7 ± 0.13	31.7 ± 0.08	31.6 ± 0.03	0.885
Hb (g dl ${}^{-1}$ )	10.6 ± 0.09	10.6 ± 0.04	10.6 ± 0.04	10.5 ± 0.07	0.807
MCV (%)	111.5 ± 1.69	112.9 ± 1.86	113.1 ± 0.6	112.2 ± 1.99	0.894
MCH (%)	35.9 ± 0.24	35.6 ± 0.87	35.7 ± 1.11	36.2 ± 0.92	0.964
MCHC (%)	33.6 ± 0.20	33.5 ± 0.15	33.6 ± 0.12	33.7 ± 0.08	0.860

Mag represents maggot meal: Mag-0 is the control diet (having 100 % soybean meal and 0 % maggot meal), whereas the other diets are Mag-10 (containing 90 % soybean meal and 10 % maggot meal), Mag-20 (having 80 % soybean meal and 20 % maggot meal) and Mag-30 (having 70 % soybean meal and 30 % maggot meal).

**Table 12:** Impact of different substitution levels of maggot meal replacement of soybean meal on the organoleptic parameters in broilers.

Group	Mean ± SE				
	Taste	Tenderness	Juiciness	Color	Flavor
Mag-0	2.37 ± 0.07	2.35 ± 0.01	2.47 ± 0.03	2.55 ± 0.02	2.78 ± 0.03
Mag-10	2.40 ± 0.12	2.37 ± 0.03	2.51 ± 0.04	2.50 ± 0.04	2.76 ± 0.02
Mag-20	2.33 ± 0.14	2.64 ± 0.17	2.50 ± 0.02	2.52 ± 0.03	2.74 ± 0.03
Mag-30	2.36 ± 0.09	2.26 ± 0.40	2.46 ± 0.05	2.50 ± 0.02	2.78 ± 0.04
p value	0.973	0.173	0.682	0.458	0.807

Mag represents maggot meal: Mag-0 is the control diet (having 100 % soybean meal and 0 % maggot meal), whereas the other diets are Mag-10 (containing 90 % soybean meal and 10 % maggot meal), Mag-20 (having 80 % soybean meal and 20 % maggot meal) and Mag-30 (having 70 % soybean meal and 30 % maggot meal).

## 4 Discussion

Several research studies have observed that the inclusion of HMM as a substitute for SBM, particularly at levels starting from 10 %, leads to notable decreases in growth performance among broilers (Khan et al., 2016). This implies that the incorporation of HMM into poultry diets might result in diminished feed consumption and reduced weight gain in broiler chickens. In the current investigation, a reduction in feed consumption was observed with increasing levels of HMM inclusion, particularly at 20 % and 30 %. This discovery supports the hypothesis suggested by Marono et al. (2017), indicating that feed color may influence the feed consumption in broilers, although this factor alone might not entirely explain the observed reduction. Further research is warranted to elucidate the underlying mechanisms. The darker-brown color of insect meals, including HMM, might be less attractive to the broilers compared to the lighter hue of SBM. However, it is essential to recognize that color might not be the sole determinant of feed intake. Moreover, previous studies (Longvah et al., 2011; Sánchez-Muros et al., 2014) have linked decreased feed intake to the high chitin content present in insect-based diets.

Chitin, a prominent component in insect exoskeletons, poses challenges to monogastric animal digestion because of its restricted digestibility, which could potentially impact protein digestibility. However, we did not assess the chitin content in the HMM. Nonetheless, it is essential to consider these factors when evaluating the influence of HMM on broiler growth performance. Research conducted by Józefiak and Engberg (2017) highlighted the challenges posed by insect protein to intestinal absorption in broilers. The challenge could stem from insects potentially acting as a reservoir of biological properties capable of influencing the gut microbial community. In a study by Khan et al. (2016), higher weight gains and dressing percentages were reported in response to reduced feed intake with a higher inclusion (30 %) of SBM replacement with HMM, which is consistent with our findings. Subsequently, M. Khan et al. (2018) reported lower feed intake and FCR but higher weight gains and dressing percentages with a 60 % replacement of SBM with HMM in another study. Taken together, these findings suggest that the use of insect protein, such as HMM, as a substitute in broiler diets could influence growth performance. The interplay between insect meal composition, nutrient absorption and gut microbiota underscores the need for further research to fully grasp these dynamics to draw conclusions regarding a better level of HMM for poultry diets. By unraveling these complexities, we can develop strategies to enhance the efficiency and sustainability of poultry production while ensuring optimal animal health and performance.

In our study, no significant differences were noted in the nutrient digestibility parameters of the broilers. However, there was a trend towards improved digestibility of various nutrients, including dry matter, crude protein, crude fiber, ash, ether extract and nitrogen-free extract, with higher levels of replacements. This aligns with previous findings suggesting that chitin, found in insect meal, could potentially hinder protein digestibility and overall dry matter digestion. However, research suggests that whole *Hermetia illucens* meals, whether they are fully intact or partially defatted, is a superior source of digestible amino acids and energy for broiler chickens, regardless of the presence of chitin. However, other studies, such as those by M. Khan et al. (2018) and Khan et al. (2016), have reported significant differences in nutrient digestibility and AME when replacing SBM with HMM, particularly at higher substitution rates.

In our study, hematological values did not vary significantly. These results are in contrast with previous findings by Shah (2020) and Elahi et al. (2020), who similarly reported no statistical differences in blood profiles where HMM was used as a substitute for SBM in broilers and layers, respectively. In contrast, Reda et al. (2023) found both linear and quadratic rises in hematological values in broilers when substituting 60 % SBM with HMM. These inconsistencies may be associated with experimental protocols, dietary compositions or the unique characteristics of the animal. Further investigation is needed to comprehensively analyze the factors influencing hematological profiles in response to HMM dietary alterations. Our study contributes to this understanding by demonstrating that incorporating HMM into the diet of broiler chicks did not adversely affect their hematological parameters. Specifically, we observed no significant changes in red blood cell count, white blood cell count, hemoglobin levels, packed cell volume or platelet count. Similarly, serum analytes did not exceed the physiological range, indicating no negative impact on these health-related parameters. These findings suggest that the substitution of SBM with HMM in broiler diets does not have detrimental effects on hematological parameters. However, it is essential to consider the potential variability in responses across different studies and to conduct further research to elucidate the underlying mechanisms and to optimize the use of HMM in poultry diets. Overall, our study adds valuable insights to the growing body of literature on the use of alternative protein sources in poultry nutrition.

In our study, incorporating HMM from 10 % to 30 % had no discernible effect on the organoleptic properties of meat, aligning with research by Cullere et al. (2018), which emphasizes consumer acceptance of insect-based diets in livestock production. Overcoming cultural barriers and ensuring safety are paramount, especially considering the importance of sensory qualities in meat products. The impact of insect meal on poultry meat may vary, but consistent findings, as observed in studies by Khan et al. (2016) and M. Khan et al. (2018), indicate no adverse effects on sensory attributes. This suggests the potential for incorporating insect-based ingredients into poultry diets without compromising consumer acceptance.

## 5 Conclusions

The findings of our study indicate that maggot meal is a valuable protein source for broiler birds, with no adverse effects observed even at replacement levels of up to 30 % in the diet. Substituting soybean meal with maggot meal did not compromise production efficiency, suggesting successful integration of maggot meal into broiler diets. This demonstrates the potential for maggot meal to serve as a sustainable alternative to soybean meal in broiler chicken production, supporting both economic and environmental objectives.

## Data Availability

the data sets used in this paper are available upon request.
